# Synthesis of Mixed-Phase TiO_2_–ZrO_2_ Nanocomposite for Photocatalytic Wastewater Treatment

**DOI:** 10.3390/toxics11030234

**Published:** 2023-02-28

**Authors:** Pooja Kumari, Rajib Saha, Gaurav Saikia, Aditya Bhujel, Mahua Gupta Choudhury, Pravin Jagdale, Samrat Paul

**Affiliations:** 1 Advanced Materials Research and Energy Application Laboratory (AMREAL), Department of Energy Engineering, North-Eastern Hill University, Shillong 793022, India; 2 Department of Applied Science and Technology, Politecnico di Torino, 10129 Turin, Italy

**Keywords:** photocatalysis, titanium, zirconium, degradation, Eosin Yellow, pollutant

## Abstract

The use of TiO_2_ nanoparticles for photocatalysis for the degradation of organic dyes under UV light for wastewater treatment has been widely studied. However, the photocatalytic characteristics of TiO_2_ nanoparticles are inadequate due to their UV light response and higher band gap. In this work, three nanoparticles were synthesized: (i) TiO_2_ nanoparticle was synthesized by a sol-gel process. (ii) ZrO_2_ was prepared using a solution combustion process and (iii) mixed-phase TiO_2_–ZrO_2_ nanoparticles were synthesized by a sol-gel process to remove Eosin Yellow (EY) from aqueous solutions in the wastewater. XRD, FTIR, UV-VIS, TEM, and XPS analysis methods were used to examine the properties of the synthesized products. The XRD investigation supported the tetragonal and monoclinic crystal structures of the TiO_2_ and ZrO_2_ nanoparticles. TEM studies identified that mixed-phase TiO_2_–ZrO_2_ nanoparticles have the same tetragonal structure as pure mixed-phase. The degradation of Eosin Yellow (EY) was examined using TiO_2_, ZrO_2_, and mixed-phase TiO_2_–ZrO_2_ nanoparticles under visible light. The results confirmed that the mixed-phase TiO_2_–ZrO_2_nanoparticles show a higher level of photocatalytic activity, and the process is accomplished at a high degradation rate in lesser time and at a lower power intensity.

## 1. Introduction

The fast growth of the textile industry has led to the build-up of many organic pollutants, with dye deposition in water bodies being one of the most problematic instances. The ecology suffers from both the direct and indirect effects of aquatic pollution [1]. Photocatalysis is a potential method for safeguarding the aquatic environment, since it may oxidize small amounts of organic pollutants in water [2]. The breakdown of organic contaminants and colours in wastewater is one of the most promising applications of photocatalysis, which uses light as a source to speed up chemical processes that are light-driven. The second largest polluting industry is thought to be the textile industry. Solid and liquid waste are the pollutants that are produced as a result of the manufacturing and consumption processes [3]. Dye is regarded as the main cause of pollution in this sector [4]. The usual categories for these contaminants are dyes [5], dissolved solids, and high chemical oxygen demand (COD) values [6]. Over 9000 dyes have been made in the colour index, according to various research investigations, and over 800,000 tons of dyes are produced annually. Due to their aromatic structure and lack of biodegradability, these dyes and their intermediates are the main water pollutants. They are potentially hazardous and poisonous, endangering both humans and the environment [7]. The primary reason for the interest in photocatalysts can be credited to their special structure as an anisotropic—one for the main advantageous properties for charge separation in photocatalytic applications [8]. The effectiveness of photocatalysis mostly depends on the decrease potential, density, and life-span of the photoinduced electrons in the semiconductors [9]. The vital factor while designing the photocatalysis materials for water treatment is the reactivity of the environment with these materials [10]. In the past two decades, several oxide and sulphide semiconductors, including TiO_2_, ZnO, WO_3_, SrTiO_3_, ZnS, and CdS, have been employed as photocatalysts for environmental regulation and a variety of chemical processes [11]. Using ZnO and TiO_2_–based nanomaterials, [12,13], Cheng et al. [14] and Ren et al. [15] have demonstrated the photocatalytic degradation of organic dyes. ZrO_2_ has been employed as a photocatalyst in a number of chemical reactions due to its extraordinarily wide band gap value Eg and strong negative conduction band potential [16]. Because nanomaterials have a greater surface ratio and shape-dependent features [17], a high rate of reaction [18] that is simple to remove, and do not create by-products, for these and other reasons, they are often used in photocatalysis processes. A bibliometric technique based on SCI databases was used to investigate the advancement of Nanomaterials and Nanotechnologies (NNs) in wastewater treatment from 1997 to 2016, and the results showed that the use of NNs in the photocatalytic reaction was progressing toward a more intricate and sophisticated classification in the spatial structure [19]. TiO_2_ is one of the most often used photocatalysts due to its optical, electrical, and catalytic properties. Anatase, rutile, and brookite are the three distinct phases of TiO_2_, which can be either crystalline or amorphous [20]. TiO_2_ is getting closer to being a superior semiconductor for photocatalysis because of its high degree of stability and safety for both people and the environment. TiO_2_ photocatalysis works better than other conventional processes and may be utilized, among other things, for “Treatment at Source” in wastewater treatment facilities, stormwater reuse, and groundwater reclamation [21]. There was a study on the synthesis of novel V_2_O_5_ bifunctional photocatalysts, and the study exhibited that these bifunctional photocatalysts were shown to be a promising and proficient photocatalytic material for both advanced oxidation and reduction processes [22]. Several scientists have doped or combined other metals with TiO_2_ nanoparticles to produce photocatalytic activity. There was a report on the synthesis of mediator Z-scheme heterojunction for photocatalysis for pollutant oxidation in water that acquires the production of constituent semiconductors with desire [23]. On the other hand, the direct Z-scheme photocatalytic mechanism has been slowly replaced by the new S-scheme heterojunction mechanism [24]. Polisetti et al. reported on the synthesis of TiO_2_, ZrO_2_, and ZrO_2_–TiO_2_ mixed oxides using the solution combustion process and observed a higher level of photocatalytic activity. When compared to commercial TiO_2_, ZrO_2_ showed a band gap of 3.5 eV, which made it a potential photocatalytic material; ZrO_2_–TiO_2_ mixed oxides were tested for the degradation of four anionic dyes [25]. However, a research group evaluated the photocatalytic activity of bimetallic ZnO–CuO hetero-nanocomposite found that the synthesized nanocomposite showed a good stability as a photocatalyst and its reusability [26]. Three primary goals of this work are to manufacture TiO_2_ using the sol-gel method, ZrO_2_ using the solution combustion method, and mixed-phase TiO_2_–ZrO_2_ nanocomposite using the sol-gel method. In addition, our aim is to investigate the photocatalytic activity levels for the degradation of Yellow Eosin dye.

## 2. Materials and Methods

### 2.1. Chemicals and Reagents Used

The chemicals and reagents used to carry out the synthesis of nanocomposites were Titanium tetraisopropoxide (TTIP) purchased from Central Drug House (P) Ltd., Dahej, India, Nitric Acid (HNO_3_) from Fischer Scientific, Zurich, Switzerland, Isopropanol from Sisco Research Laboratory Pvt. Ltd., Mumbai, India, Zirconium (IV) oxynitrate hydrate (H_2_N_2_O_8_Zr) from Sigma-Aldrich, Saint Louse, USA and methanol from Merck Life Science Pvt. Ltd., Bengaluru, India, EOSIN Y (dye) (C_20_H_6_Br_4_Na_2_O_5_) was purchased from Sisco Research Laboratories Pvt. Ltd., Mumbai, India, and was used as a pollutant in the photocatalytic activity. Further, the chemicals were used without undergoing any purification.

### 2.2. Synthesis of TiO_2_, ZrO_2_, and Mixed Phase TiO_2_–ZrO_2_ Nanocomposite

#### 2.2.1. Synthesis of TiO_2_

TiO_2_ was produced using the sol-gel process [27] with further modification. Prior to combining TTIP and isopropanol to create titania sol, the mixture was continuously swirled in a beaker. After 15 min, the mixture received 1 mL of HNO_3_.Titania gel was produced by letting the solution stand for 12 h while being continuously agitated, and then calcining it for 1 h at 730 °C in a muffle furnace.

#### 2.2.2. Synthesis of ZrO_2_

ZrO_2_ was created using the solution combustion process. Zirconium (IV) Oxynitrate Hydrate and warm distilled water were combined to generate the precursor solution, which was then heated to 80 °C for an hour while being constantly stirred. After an hour, the resulting solution was mixed 1:1 with Methanol and heated in a muffle furnace for an hour at 650 °C to generate the necessary nanomaterial.

#### 2.2.3. Synthesis of Mixed-Phase TiO_2_–ZrO_2_ Nanocomposite

The sol-gel method was used to produce a nanocomposite of mixed-phase TiO_2_ and ZrO_2_. In a beaker, synthesized ZrO_2_ was added after mixing TTIP and isopropanol together while stirring constantly. After 15 min, the mixture received 1 mL of HNO_3_. Titania gel was produced by letting the solution stand for 12 h while being continuously agitated, and then calcining it for 1 h at 730 °C in a muffle furnace.

## 3. Characterization Technique

The internal structure and shape of the produced nanoparticles were investigated using a transmission electron microscope (TEM) model JEM-100CX II from JEOL Pleasanton, CA, USA. Fourier Transform Infrared Spectroscopy (FTIR, IMPACT 410, and NICOLET, Tramelan, Switzerland was used to investigate the chemical characteristics of the produced samples, and X-ray Diffraction analysis (XRD, D8 Advance Bruker, Leipzig, Germany) was used to study the crystallinity of the samples. Thermo Scientific’s ESCALAB Xc+ was used for the X-ray photoelectron spectroscopy (XPS), while Aligent, California, USA’s Cary 100 UV-vis spectrometer was used for the UV analysis.

TiO_2_, ZrO_2_, and mixed-phase TiO_2_–ZrO_2_ were investigated as photocatalysts under the visible light conditions of Eosin Yellowish dye. We initially created dye solutions in order to conduct the experiment. Eosin Y is typically used in concentrations of 0.5–1%, i.e., 1 mL of Eosin Yellowish dye is used to colour 100 mL of distilled water. Photocatalysts composed of 10 mg each of TiO_2_, ZrO_2_, and mixed TiO_2_–ZrO_2_ were added to beakers holding 500mL of Eosin Yellowish dye solution. The aqueous solution of Eosin Yellow exhibited a maximum excitation peak at 512 nm while the emission spectrum showed a peak at 523 nm. The equilibrium condition between absorption and desorption was achieved by magnetically stirring the dye/catalyst solutions for an hour in the dark. The photocatalytic investigations were conducted under a 200 W Xenon lamp using visible light (illuminance of 940 lux). An amount of 2 mL of the solution was immediately withdrawn from the top surface at intervals of 60 min. We were able to calculate the residual concentrations of the Eosin Yellowish dye using UV–vis spectra.

## 4. Results and Discussion

### 4.1. XRD

**Figure 1:**
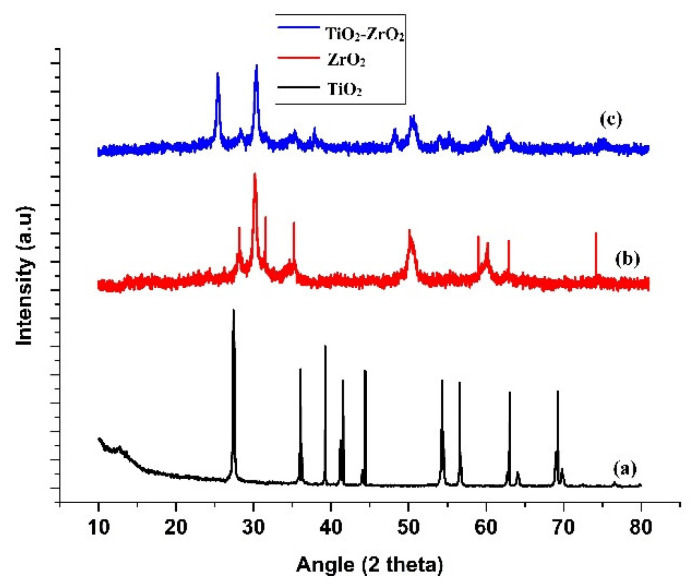
XRD patterns for (**a**) TiO_2,_ (**b**) ZrO_2,_ and (**c**) mixed-phase TiO_2_–ZrO_2_ nanocomposite.

Figure 1 shows the X-ray Diffraction Spectra (XRD) for the prepared samples that were calcined at various calcination temperatures.

The spectra’s high intensity peaks demonstrate the sample’s great crystallinity. Figure 1a shows the XRD pattern for TiO_2_ and shows the recognizable peak of TiO_2_ with a tetragonal structure that closely matches JCPDS CARD NO. 00-021-1272, including (101), (004), (200), (105), (211), (204), (116), (220), and (215). Figure 1b displays the different ZrO_2_ peaks (110), (111), (200), (220), and (302), whereas JCPDS CARD NO. 98-004-1010 designates the monoclinic phase of the ZrO_2_ crystal. All of the TiO_2_ and ZrO_2_ peaks are present in the mixed-phase TiO_2_–ZrO_2_ in Figure 1c. However, there are some minor peaks—as observed in the graph of the nanocomposite—that also confirm the formation of the core-shell structures. From the diffraction patterns of the nanocomposite, it can be observed that there are major peaks for TiO_2_ and ZrO_2_, confirming the presence of these compounds. Moreover, the results affirm that the nanocomposite has a high level of purity and the decomposition processes for TiO_2_ and ZrO_2_ have yet to start. Scherrer’s relation was used to calculate the average crystallite size (D) of the monoclinic ZrO_2_ nanoparticles as well as the tetragonal TiO_2_ nanoparticles. The tetragonal TiO_2_, monoclinic ZrO_2_, and mixed-phase TiO_2_–ZrO_2_ NPS’s crystal sizes were determined to be 13.55, 7.09, and 5.65 nm, respectively.

### 4.2. TEM

**Figure 2:**
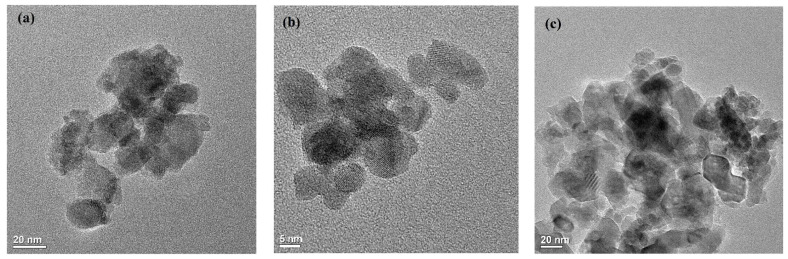
TEM micrographs of (**a**) TiO_2_, (**b**) ZrO_2_, and (**c**) mixed-phase TiO_2_–ZrO_2_ nanoparticles.

The inner morphology of TiO_2_, ZrO_2,_ and mixed-phase TiO_2_–ZrO_2_ nanoparticles were studied using TEM, as shown in Figure 2a–c. The micrographs established the crystalline structure of the nanoparticles synthesized. The average particle size of the synthesized nanoparticles was determined from the high-resolution TEM analysis. The average particle sizes for TiO_2_and ZrO_2_ were found to be around 43 nm and 45 nm, respectively. For the mixed-phase TiO_2_–ZrO_2_ nanoparticles, the average particle size was found to be around 41 nm. The micrographs confirmed that the nanocomposites with insulating TiO_2_ shells had better structural stability. The TEM analysis showed that the mixed-phase TiO_2_–ZrO_2_ nanocomposite had ultra-small nanoclusters with a narrow particle size distribution.

### 4.3. XPS

**Figure 3:**
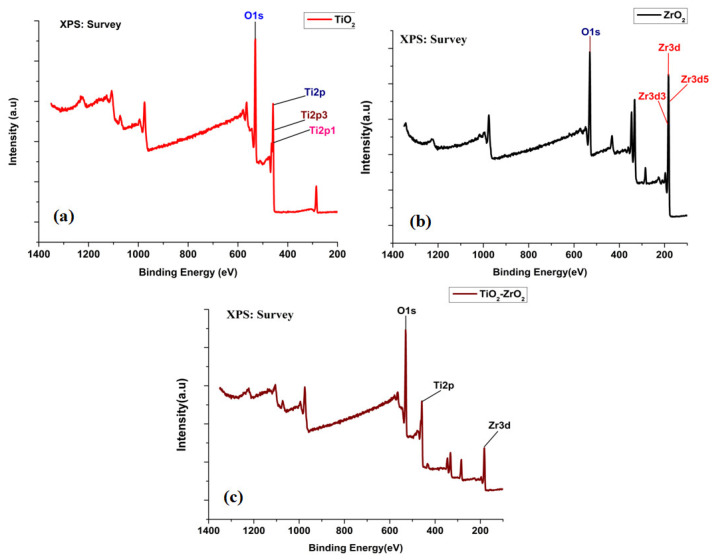
XPS spectra of (**a**) TiO_2_, (**b**) ZrO_2_, and (**c**) mixed-phase TiO_2_–ZrO_2_ nanoparticles.

Figure 3a–c shows the high-resolution XPS spectra of the core levels of the three samples. XPS data were obtained as spectra that plot binding energy (eV) on the *X*-axis vs. measured photoelectron counts (intensity) on the *Y*-axis. The maxima in the O1s binding energy region are characteristic to the O^−2^ of TiO_2_. The doublet Ti2p3/2 (binding energy 459 eV) and Ti2p1/2 (binding energy 465 eV) arise from spin orbit splitting. These peaks are consistent with the Ti^4+^ in the TiO_2_ lattice. The binding energy of O1s is determined to be 530.4 eV. The mound shape of the O1s spectrum shows the presence of embedded surface species, which were determined through peak deconvolution to have four peaks at 530.7, 531.9, 532.9, and 534.0 eV [28]. The presence of Ti^3+^ and Ti^4+^ oxidation states was confirmed from the position of the Ti2p3/2peak at 459.1 eV and the shoulder at 458.0 eV, which is in good agreement with previous reports [29,30]. The peak at 530.4 eV is assigned to oxygen bound to tetravalent Ti ions. Now, as observed in the optical analysis, the band gap of the TiO_2_ nanoparticle (3.22 eV) decreased to 2.70 eV. The FWHM for the Ti2p3/2 peak was determined to be 4.29 eV.

The spectrum of ZrO_2_ is shown in Figure 3b. The BE maximum for the Zr3d core-line was found at 186eV, whereas the BE for Zr3d3/2 and Zr3d5/2 were 182 eV and 184 eV, respectively. The binding energy of O1s was determined to be 530.4 eV. The detected relative BE shifts are most likely due to different surface electrostatic charges. The BE maximum for the Zr3d core-line was found at 182–186 eV, in agreement with other data reported in the literature [31,32]. The FWHM values were found to be 2.61 eV, 5.12 eV, and 1.17 eV, respectively. The spectrum can be fitted with two doublets—the greater one at 186 eV and the smaller at 182 eV. A solid reduction with stoichiometry variations ≥ 2% in the near-surface region can be excepted from the XPS measurements, Zr:O and tetragonal ZrO_2_ ratios [33]. The optical band gap for ZrO_2_ using XPS analysis was found to be 6.8 eV.

Two main peaks, one for zirconium (Zr3d) and one for titania (Ti2p), were detected in the XPS spectra (Figure 3c). The peak shift dependence on the Ti-content was observed in the spectra for the mixed-phase TiO_2_–ZrO_2_ nanoparticles, indicating Ti-O-Zr bond formation. The main peaks of the Ti2p doublet are presented, and the location of stronger peak Ti2p at 459 eV is in good agreement with other reports [34]. The extension at a higher binding energy in the O1s peak at 531 eV is barely usable because of the occurrence of a non-negligible amount of oxygen that was involved in the carbonaceous contamination [35]. The value of titanium indicates that it is present in the +4 oxidation state [36]. The Zr3d photoelectron peaks were detected at 184.2 and 186.4 eV, conforming to Zr3d5/2 and Zr3d3/2, respectively, which are assigned to the +4 oxidation state of zirconium as reported in earlier reports [37,38]. For TiO_2_–ZrO_2_ nanoparticles, the band gap was found to be 2.70 eV and 6.7 eV, which could be attributed to TiO_2_ and ZrO_2_, respectively. The FWHM values, as obtained from theTi2p and Zr3d core-lines, were found to be 5.11 eV and 4.64 eV, respectively. The assignment of the corresponding signals observed in the XPS spectra for three elemental species, titanium, zirconium, and oxygen, were identified in the nanoparticles. Figure 3 represents the Zr 3d, Zr 3p3/2, Zr 3d5/2, Ti 2p3/2, O 1s, Ti 2p1/2, and Ti 2p XPS spectra (core lines). This analysis confirmed the declared empirical formulas and demonstrated the high purity of the samples.

### 4.4. FTIR

**Figure 4:**
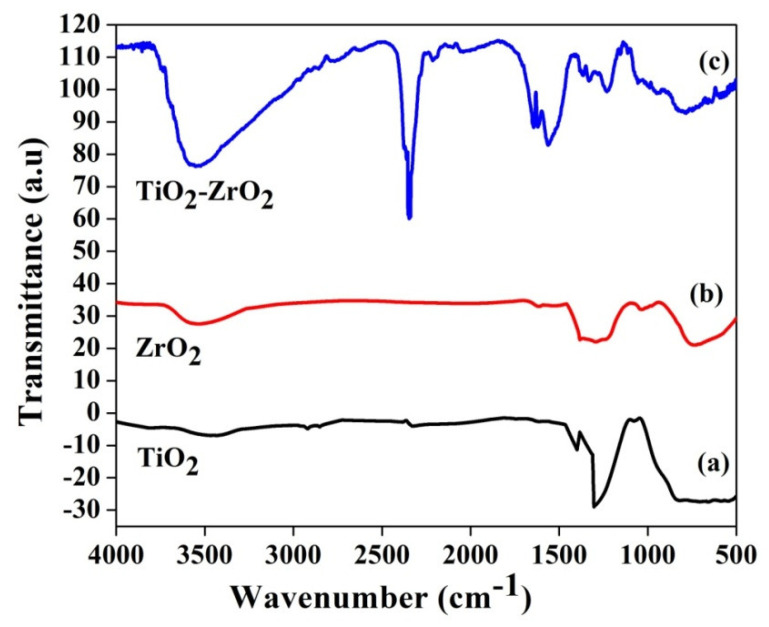
FTIR spectra of (**a**) TiO_2_, (**b**) ZrO_2_, and (**c**) mixed-phase TiO_2_–ZrO_2_ nanocomposite.

FTIR analysis was done to thoroughly assess the metal–oxygen and carbon–oxygen bonding in the produced TiO_2_, ZrO_2_, and mixed-phase TiO_2_–ZrO_2_ samples obtained in the 500–4000 cm^−1^ range, as shown in Figure 4a–c. Ti-O and/or Ti-O-Ti bonds are responsible for the observed transmittance bands in the range 800–500 cm^−1^, as shown in Figure 4a [39]; also detected at these frequencies and ascribed to an O-H bending mode are transmittance bands at 1431 and 1648 cm^−1^. Additionally, in the wavenumber range of 3650–3000 cm^−1^, large transmittance bands were observed that were physically adsorbed water on the surface of the TiO_2_ nanoparticle [40]. Two transmittance bands were seen at 2029 and 2345 cm^−1^, which are the combination bands in TiO_2_ nanoparticles [41,42,43,44]. The absorption peak at 755 cm^−1^ in the 500-850 cm^−1^ range relates to the vibration of the Zr-O bond, and a wide peak of about 3500 cm^−1^ relates to the presence of the stretching vibration of hydroxyl groups on the surface of the samples [40]. According to Figure 4b, the bending vibration of the C−H bands is what caused the weak absorption bands at 1036 cm^−1^ and 1291 cm^−1^. The 632 and 790 cm^−1^ absorption bands in Figure 4c may be caused by Zr-O and Ti-O interatomic vibrations. In the O-H hydroxyl group, the water molecules on the surface of the nanoparticles stretched, which caused the high absorption peaks in the spectra in the 3500–3880 cm^−1^ area [45].

### 4.5. Determination of Band Gap Using the Kubelka–Munk Function

**Figure 5:**
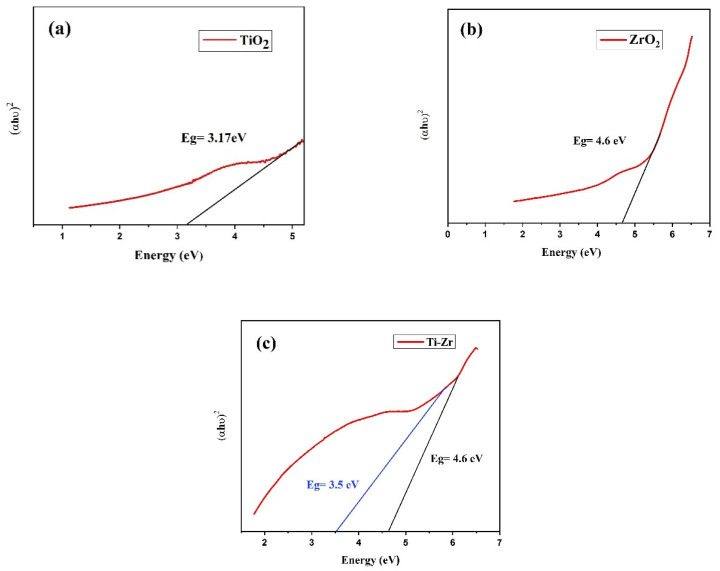
Kubelka–Munk plot of (**a**) TiO_2_, (**b**) ZrO_2_, and (**c**) mixed-phase TiO_2_–ZrO_2_ nanoparticles.

The measurement of diffuse reflection (DR) using a UV-Vis spectrophotometer is a normal technique used to determine the optical band gap of nanomaterials. The Kubelka–Munk function was used to determine the band gap for TiO_2_, ZrO_2_, and mixed-phase TiO_2_–ZrO_2_ separately after the diffuse reflectance spectra (DRS) for these materials were obtained. The reflectance results for all of the three samples shown in the Figure 5 were taken as a function of the incident radiation wavelength range (300–800 nm) obtained from DRS. Figure 5 illustrates the Kubelka–Munk plot for the TiO_2_, ZrO_2_, and mixed-phase TiO_2_–ZrO_2_ nanocomposite. The Kubelka–Munk function was used to determine the band gap energies of the generated nanoparticles in accordance with the following equation.

$$F(R)=\frac{{\left(1-R\right)}^{2}}{2R}$$

R implies diffuse reflectance because [F(R) E]^n^ = A(E − E_g_), where n indicates whether the band gap is direct (n = 2) or indirect (n = 1/2), F(R) is the K-M function, and A specifies constant semiconductor characteristics. In the Figure 5 below, it shows how the experimental optical band gaps for TiO_2_, ZrO_2_, and mixed-phase TiO_2_–ZrO_2_ nanoparticles were obtained by extrapolating the linear section of the curve [F(R)E]^2^ against the photon energy (E). The band gap was found to be 4.72 eV at an absorption edge of 326 nm for TiO_2_, 4.6 eV at an absorption edge of 263 nm for ZrO_2_. Whereas for mixed-phaseTiO_2_–ZrO_2_ nanocomposite both materials presences could be recorded with band gaps at 3.5 and 4.6 eV at an absorption edge of 326 nm and 263 nm, respectively. As a result, we can conclude that adjusting the optical band gap is appropriate for a number of uses, including photovoltaics, photocatalysis, and thermoelectric applications [46,47].

### 4.6. Photocatalytic Activity

The produced samples’ photocatalytic activity levels were investigated in the solar simulator with light intensities of 940 lux and a 200 W Xenon lamp as the light source. Eosin Yellowish was the dye used to check the samples’ photocatalytic activity (C_20_H_6_Br_4_Na_2_O_5_).

Initially, 1 mL of distilled water (w/v) was dissolved to create the dye solution. Separately, 50 mL of dye solution was added after 10 mg of nanoparticles. The photocatalytic investigations were conducted under a 200 W Xenon lamp using visible light (an illuminance of 940 lux). The number of molecules that light interacts with affects how much of it is absorbed. After being constantly stirred for 15 min, both nanoparticle solution mixes were exposed to light for 60 min.

**Figure 6:**
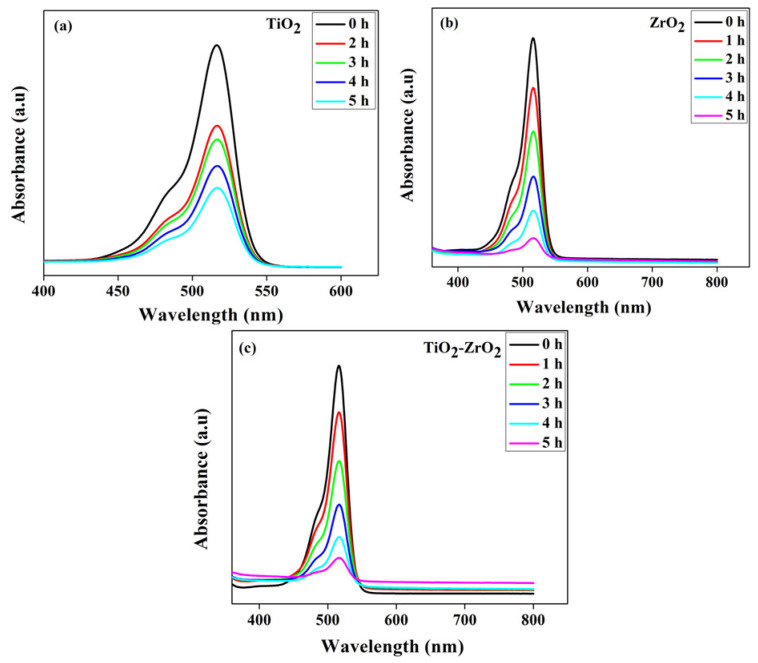
Using (**a**) TiO_2_, (**b**) ZrO_2_, and (**c**) mixed-phase TiO_2_–ZrO_2_ nanocomposite, temporal variations in the absorbance spectra of Eosin Yellowish were observed.

The dye solutions were continuously mixed and put under a light source to check for any colour changes. The Eosin Yellow dye deteriorated, as seen by the dye solution’s gradual shift in hue from a rich pink to a colourless tint. The dye degradation was investigated using UV-visible absorption spectroscopy, in presence of both nanoparticles in dye solution. A drop in absorbance over the duration of light exposure [19] was used to establish that the dye degraded in the presence of both nanoparticles. The Eosin Yellowish absorption spectra showed a large absorption peak at 517 nm. The absorbance value fell with time because of the dye degradation brought on by UV light irradiation, as seen in the graph in Figure 6. The sample’s spectral response was examined using an Aligent USA UV model Cary 100 Uv-Vis spectrometer. The following calculation was used to compute the proportion of Eosin Yellowish that had been degraded:

Degradation% = (1 − C/C_0_) × 100

where C_0_ is the Eosin Yellowish concentration before illumination and C is the concentration following a certain amount of irradiation [46]. In the dye degradation experiment utilizing TiO_2_, ZrO_2_, and mixed-phase TiO_2_–ZrO_2_ nanocomposite, the relative percentage of degradation for each was 64.74% for TiO_2_, 86.14% for ZrO_2_, and 87.87% for mixed-phase TiO_2_–ZrO_2_ nanocomposite, according to the data. As a consequence, we observed that the pollutant’s absorption value decreased over time when exposed to sunlight or a solar simulator.

### 4.7. Kinetic Study for the Degradation of Dye

The photocatalytic capabilities of the samples were examined in the degradation of Eosin Yellow as a dye.

The model appears to suit the first-order kinetics according to the following formula:

$$\mathrm{Log}\;(\frac{C_{0}}{C})=-kt$$

where *k* is the dye photodegradation rate constant, and C_0_ and C are the dye concentrations before and after exposure to light for time *t* (min), respectively.

**Figure 7:**
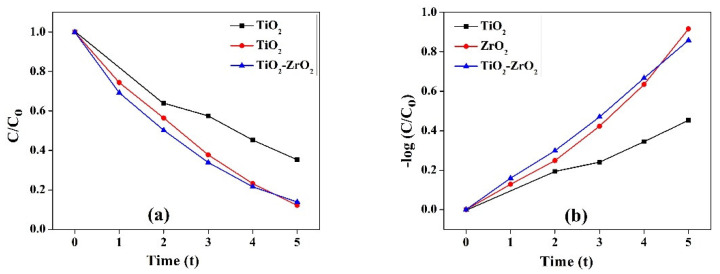
Kinetic study of (**a**) log C/C_0_ vs. time and (**b**) C/C_0_ Vs. time for TiO_2_, ZrO_2_, and mixed-phase TiO_2_–ZrO_2_ nanocomposite.

The rate constants were obtained by plotting ln C/C_0_ Vs. time. For the nanocomposite, the rate constants were 0.088021 min^−1^ for TiO_2_, 0.179243 min^−1^ for ZrO_2_, and 0.170965 min^−1^ for the mixed-phase TiO_2_–ZrO_2_. The dye degradation rate constants under visible light exposure are shown in Figure 7. The photocatalytic studies increased UV light absorption and widened the visible light sensitivity zone during photocatalytic activity. It is noteworthy that the plot’s exponential existence of absorbance vs. time (log C/C_0_ vs. time/C/C_0_ vs. time) corroborated the pseudo first-order kinetics of the reaction, since the convergence of the dye concentration at different periods corresponds to the dye solution’s absorbance.

Under photocatalytic conditions, the TiO_2_, ZrO_2_, and mixed-phaseTiO_2_–ZrO_2_ nanocomposite dissolved 64.74%, 86.14%, and 87.87%% of the dye after 60 min. The dye solution’s absorbance is related to the dye concentration’s convergence at different times; therefore, the reaction’s pseudo-first-order kinetics is supported by the exponential decay of absorbance with time.

### 4.8. Proposed Model

**Figure 8:**
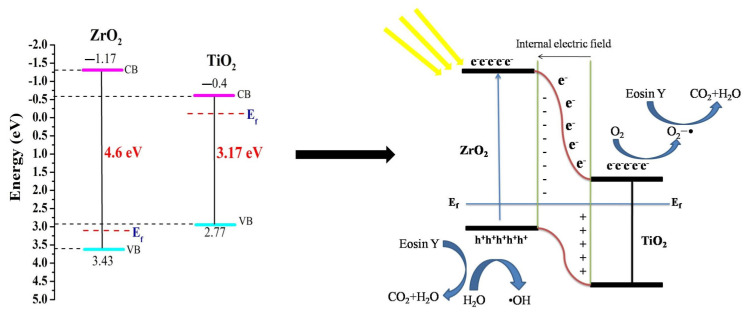
Eosin Y photocatalytic degradation mechanism over TiO_2_, ZrO_2_, and mixed-phase TiO_2_–ZrO_2_ nanocomposite exposed to visible light.

The following is an explanation for the enhanced photodegradation of Eosin Y brought on by the TiO_2_ and ZrO_2_ samples: It has been determined that the metal atom composite causes the formation of new phases scattered throughout the TiO_2_, trapping the photogenerated species (e^−^/h^+^) by preventing their recombination [48]. In essence, the newly produced energy levels serve as electron capture centres, aiding in the separation of the photogenerated e^−^/h^+^ couples [49]. However, the presence of too many flaws encourages recombination, which reduces photoactivity. Due to the energy level of TiO_2_ for both VB and CB being contained inside the band gap of ZrO_2_, the separation of e^−^/h^+^ between TiO_2_ and ZrO_2_ in the mixed oxide may be accomplished. Therefore, the exciting electrons of ZrO_2_ and TiO_2_ migrate from their respective VB to CB under the influence of a photon with energy greater than the band gap energy of the semiconductors, producing e^−^/h^+^ pairs. Then, a portion of the photogenerated electrons (e^−^) of ZrO_2_ is grabbed by the CB of TiO_2_, while holes (h^+^) from TiO_2_ are trapped by the VB of ZrO_2_, aiding in the improvement of charge separation and reducing the recombination process [50]. As a result, when oxygen combines with the collected e^−^ from ZrO_2_ contained in TiO_2_’s CB, it produces superoxide radicals of O_2_^•−^ because of its high electron acceptor properties. On the other hand, the hydroxyl radical oxidation process for H_2_O to form hydroxyl radicals (•OH) may now be fully carried out using the h^+^ from the TiO_2_ collected by the VB of ZrO_2_ [51]. Finally, the organic molecules are broken down into simpler compounds by the superoxide (O_2_^•−^) and hydroxyl radicals (•OH), which results in CO_2_ and H_2_O being the overall by-products [52,53]. According to the hypothesized mechanism, seen in Figure 8, this process of the migration and capture of photogenerated charges caused by the presence of distributed impurities increased the e/h+ pair separation and decreased the rate of recombination.

On the other hand, light can stimulate the dye. When exposed to light, the dyes adsorbed into the powder structure had a photosensitizing effect and liberated free electrons that could penetrate the internal structure of the photocatalyst. Dye sensitization is the name of this mechanism. When exposed to photons, the dye molecules that are adhered to the photocatalyst’s surface are photoexcited from the highest occupied molecular orbital (HOMO) to the lowest unoccupied molecular orbital (LUMO) [45]. The dye then becomes a cationic radical as a result of the photoexcited electrons from the LUMO being caught by the CB of the photocatalyst. This radical may now be photodegraded to create less dangerous compounds. The oxygen in the CB of the photocatalyst traps the e^−^ to produce O_2_^•−^ and the dye’s photodegradation. Usually, the dye sensitization process takes place when dye molecules are adsorbed on the catalyst structure.

## 5. Conclusions

In conclusion, ZrO_2_ nanoparticles were produced using the solution combustion approach, whereas TiO_2_ mixed-phase TiO_2_–ZrO_2_ nanoparticles were produced using the sol-gel method. The XRD investigation supported the tetragonal and monoclinic crystal phases of TiO_2_ and ZrO_2_. The particle sizes of TiO_2_ and ZrO_2_ were determined by a TEM examination to be around 43 nm and 45 nm, respectively. The particle size for the mixed-phase TiO_2_–ZrO_2_ nanoparticles was likewise discovered to be about 41 nm. According to the UV-DRS spectroscopy, the energy gap values for TiO_2,_ ZrO_2,_ and mixed-phase TiO_2_–ZrO_2_ were found to be 3.17, 4.6, 3.5 and 4.6 eV, respectively. Additionally, for TiO_2_, ZrO_2_, and mixed-phase TiO_2_–ZrO_2_ photocatalysts under UV irradiation, the rates of Eosin Yellow dye degradation were determined to be 64.74%, 86.14%, and 87.87% within 5 h. Mixed-phase TiO_2_–ZrO_2_ was found to be the best material for photocatalytic application. The full degradation of the dye was obtained at the lowest dye concentration, the lowest pH value, at a temperature of 75 °C and a low power intensity of the UV lamp. The full degradation was achieved at a time period of 180 min.

## 6. Future Work

In this paper, we have only performed an initial study of the photocatalysis materials. We will perform the Scavenger and reusability test in our future work with the same materials. The activity and stability of the catalyst will be extensively evaluated in our future study with the same nanoparticles. Active species capture experiments will also be conducted by us in our future work.

## Data Availability

The data used to support the research findings are available from the corresponding author upon request.
